# Dataset of longitudinal analysis of tear cytokine levels, CD4, CD8 counts and HIV viral load in dry eye patients with HIV infection

**DOI:** 10.1016/j.dib.2017.01.014

**Published:** 2017-02-03

**Authors:** Praveen Kumar Balne, Rupesh Agrawal, Veonice Bijin Au, Bernett Lee, Eileen Loo, Louis Tong, Arkasubhra Ghosh, Stephen C. Teoh, John Connolly, Petrina Tan

**Affiliations:** a National Healthcare Group Eye Institute, Tan Tock Seng Hospital, Singapore; b Singapore Eye Research Institute, Singapore; c Institute of Molecular and Cell Biology, Singapore; d Duke-NUS Medical School, Singapore; e National University of Singapore, Singapore; f GROW Research Laboratory, Narayana Nethralaya Foundation, India; g Eagle Eye Centre, Singapore; h Jurong General Hospital, Singapore

**Keywords:** Dry eye disease, Cytokine profiles, HIV infection and longitudinal study

## Abstract

The data presented in this article shows the longitudinal analysis of tear fluid cytokine profiles, blood CD4 and CD8 counts and HIV viral load in 34 dry eye patients with HIV infection during the HAART therapy. Clinical samples were collected from HIV patients with dry eye disease at the time of presentation to the clinic (visit 1), three months (visit 2) and 6 months (visit 3) after the presentation. At each time point tear samples were evaluated for 41 cytokines using Luminex bead based multiplex assay and blood samples were tested for HIV viral load and CD4 and CD8 counts.

**Specifications Table**

**Table:** 

Subject area	*Biology*
More specific subject area	*Tear cytokine profiles in dry eye patients with HIV infection*
Type of data	*Table*
How data was acquired	*Luminex assay using the Milliplex*® *MAP human cytokine/chemokine magnetic bead panel -1 kit with FlexMAP 3D (Luminex*®*) platform*
Data format	*Raw*
Experimental factors	*Tear and blood samples collected from dry eye patients with HIV infection at three different time points.*
	*At each time point tears were analyzed for 41 cytokines using Luminex bead based multiplex assay and systemic CD4, CD8 counts and HIV viral loads were tested using Trucount tubes and qRT-PCR respectively.*
Experimental features	*Milliplex*® *MAP human cytokine/chemokine magnetic bead panel -1 kit (Millipore, USA) with FlexMAP 3D (Luminex*®*) platform was used for cytokine profiling in tear samples*
Data source location	*Singapore*
Data accessibility	*Data are with this article*

**Value of the data**

• In our earlier reports cytokines IFN-gamma-inducible protein 10 (IP-10, CXCL10), epidermal growth factor (EGF) and growth-regulated oncogene (GRO) levels were significantly altered in dry eye patients with human immunodeficiency virus (HIV) infection compared to immunocompetent dry eye patients at the time of presentation (visit 1/baseline) to the clinic [1] and the dataset of baseline cytokine levels and clinical parameters were reported [2].
• Here we report the longitudinal tear cytokine profiling data of HIV patients with dry eye disease over the period of 6 months with two follow ups (3 months each) and its association with the systemic CD4 (cluster of differentiation 4), CD8 counts and HIV viral loads.
• This longitudinal data will help researchers to understand the changes in tear cytokines in HIV patients with dry eyes over the time and its association with systemic and viral factors and study further on HIV associated ocular inflammation and its pathogenesis.

## 1 Data

The data presented herein was obtained from longitudinal analysis of tear cytokine profiles in 34 dry eye patients with HIV infection at 3 time points (Table 1). Systemic CD4 and CD8 counts and HIV viral loads (Table 2) were also analyzed at each time point to elucidate the association of HIV, systemic and ocular factors in dry eye disease.

## 2 Experimental design, materials and methods

The data herein was obtained from HIV patients who had complaints of symptoms related to dry eyes (foreign body sensation, dryness or roughness and irritation) with a prior informed consent and were evaluated following the guidelines of the International Dry Eye Workshop (DEWS), 2007 [3]. HIV diagnosis was confirmed by Western blot assay (Diagnostic Biotechnology HIV Blot 2.2) and the severity of the dry eye was evaluated at the time of presentation using different clinical tests as mentioned in the earlier report [1]. Thirty four HIV patients with dry eyes were enrolled for the longitudinal analysis and blood and tear samples were collected at 3 time points (at the time of presentation to the clinic (visit 1), 3 months (visit 2) and 6 months (visit 3) after the presentation). Out of 34 patients, one patient died and two patients were withdrawn from the study after the first visit. Eight patients were missed the visit 2 and two patients were missed the visit 3. At each time point tear samples were collected using Schirmer׳s strips and 10 mL of blood was collected aseptically through vein puncture and transferred into an anticoagulant ethylenediamine tetraacetic acid (EDTA) containing tube. Tear fluid was eluded from Schirmer׳s strips as mentioned earlier [1] and stored at −80 °C until cytokine analysis [4]. At each time point HIV viral load and blood CD4 and CD8 counts (Table 2) were determined by quantitative real time reverse transcriptase polymerase chain reaction (qRT-PCR) and Trucount tubes (BD Biosciences, San Jose, CA, USA) according to the manufacturer׳s instructions respectively. Schirmer׳s test was done on HIV patients at each visit (Table 2) and tear cytokine profiling was done at each time point by Luminex bead based multiplex assay with a panel of 41 analytes (Table 1) using Milliplex® MAP human cytokine/chemokine magnetic bead panel −1 kit (Millipore, USA) following the manufacturer׳s instructions.

## Appendix A Supplementary material

Table 1: Longitudinal data of 41 cytokines levels (average and minimum–maximum concentrations) in the tear film of dry eye patients with HIV infection.

.

Table 2: Longitudinal data of blood CD4 and CD8 counts and HIV viral load in dry eye patients with HIV infection.
